# Localized Spin Dimers and Structural Distortions in the Hexagonal Perovskite Ba_3_CaMo_2_O_9_

**DOI:** 10.1021/acs.inorgchem.2c01102

**Published:** 2022-07-19

**Authors:** Struan Simpson, Michael Milton, Sacha Fop, Gavin B. G. Stenning, Harriet Alexandra Hopper, Clemens Ritter, Abbie C. Mclaughlin

**Affiliations:** † Chemistry Department, University of Aberdeen, Meston Walk, Aberdeen AB24 3UE, U.K.; ‡ ISIS Experimental Operations Division, Rutherford Appleton Laboratory, Harwell Science and Innovation Campus, Didcot OX11 0QX, U.K.; § Institut Laue Langevin, 71 Avenue des Martyrs, F-38042 Grenoble Cedex 9, France

## Abstract

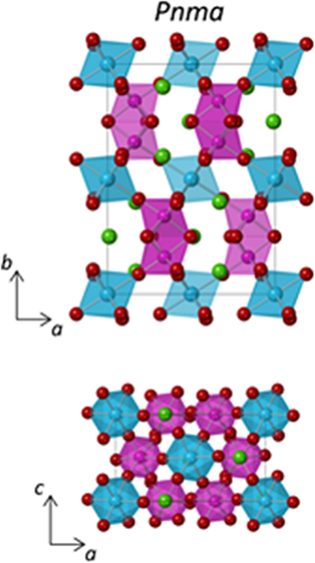

Extended solid-state materials based on the hexagonal perovskite framework are typified by close competition between localized magnetic interactions and quasi-molecular electronic states. Here, we report the structural and magnetic properties of the new six-layer hexagonal perovskite Ba_3_CaMo_2_O_9_. Neutron diffraction experiments, combined with magnetic susceptibility measurements, show that the Mo_2_O_9_ dimers retain localized character down to 5 K and adopt nonmagnetic spin-singlet ground states. This is in contrast to the recently reported Ba_3_SrMo_2_O_9_ analogue, in which the Mo_2_O_9_ dimers spontaneously separate into a mixture of localized and quasi-molecular ground states. Structural distortions in both Ba_3_CaMo_2_O_9_ and Ba_3_SrMo_2_O_9_ have been studied with the aid of distortion mode analyses to elucidate the coupling between the crystal lattice and electronic interactions in 6H Mo^5+^ hexagonal perovskites.

## Abstract 2

A novel hexagonal perovskite, Ba_3_CaMo_2_O_9_, has been synthesized. Neutron diffraction experiments, combined with magnetic susceptibility measurements, show that, in Ba_3_CaMo_2_O_9_, the Mo_2_O_9_ dimers retain localized character down to 5 K and adopt nonmagnetic spin-singlet ground states. A structural transition from *P*6_3_/*mmc* to *Pnma* symmetry is observed near 200 K, but the electronic and magnetic properties of the Mo_2_O_9_ dimers appear to be largely insensitive to the resulting distortion.

## Introduction

Transition metal oxides (TMOs) display a wealth of exotic physical phenomena due to the intrinsic coupling between their lattice and electronic degrees of freedom. In recent years, extended solid-state TMOs containing closely spaced transition metal atoms have attracted widespread interest for their ability to form clustered quasi-molecular (QM) states.^1^ Typically, these “orbital molecules” manifest as a result of orbital ordering and direct bonding between the metal atoms.^2^ A pertinent example is found in the naturally occurring mineral magnetite (Fe_3_O_4_), in which subtle contractions in the Fe^2+^–Fe^3+^ distances accompany the formation of three-site Fe^3+^–Fe^2+^–Fe^3+^ bonded trimerons.^3,4^ In general, such metal–metal bonded clusters arise when the geometrical connectivity between neighboring atoms imposes considerable overlap between the metal d orbitals; in particular, materials with edge-sharing or face-sharing polyhedra are promising in this regard,^5^ with other notable examples including TMOs such as VO_2_,^6,7^ Li_2_RuO_3_,^8,9^ and Y_2_Mo_2_O_7_.^10^ Orbital molecules are often formed in tandem with metal–insulator transitions and are characterized by spin-gapped magnetic excitations. As such, orbital molecules exhibit fundamentally interesting physical properties that may hold future applications in spintronic or orbitronic technologies.

**Figure 1:**
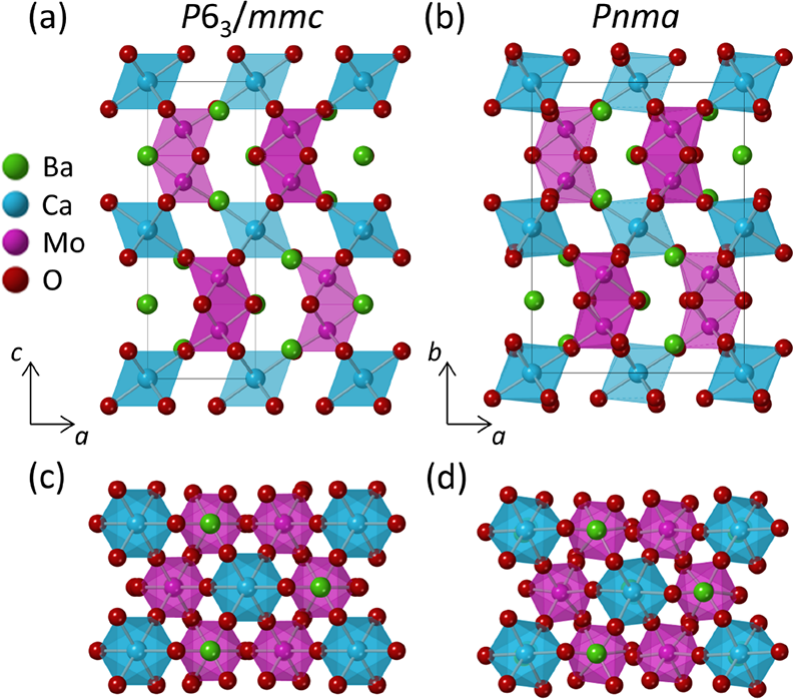
Crystal structures of Ba_3_CaMo_2_O_9_ at (a) 290 K (space group: *P*6_3_/*mmc*) and (b) 5 K (space group: *Pnma*). Projections of the *P*6_3_/*mmc* and *Pnma* structures down the long axes are provided in panels (c) and (d), respectively.

In recent years, orbital molecules have been proposed to emerge in hexagonal perovskites of the form Ba_3_*B*′Ru_2_O_9_ (*B*′ = Na;^11^ Y, In, and Lu;^12^ and Ce^13^). These materials crystallize in a six-layer hexagonal (6H) structure consisting of corner-sharing *B*′O_6_ octahedra and face-sharing *M*_2_O_9_ bioctahedral dimers (Figure 1a). The *M*_2_O_9_ dimers are characterized by short intradimer *M*–*M* distances that favor electronic interactions between the metal atoms. When *M* is a 4d/5d transition metal (such as Ru or Ir), the spatially diffuse *t*_2g_ orbitals can overlap, and with sufficiently weak Hund’s coupling, direct *M*–*M* bonds can then form between the metal atoms.^14,15^ However, some Ba_3_*B*′*M*_2_O_9_ compositions retain localized magnetic moments on the metal atoms so that, in this case, the *M*_2_O_9_ dimers are better described as unbonded cluster magnets (rather than orbital molecules).^16−19^ This is due to the strong competition between Hund’s coupling and *M*–*M* bonding in a face-sharing polyhedral geometry.^20,21^ Such geometries are idiosyncratic motifs of hexagonal perovskites, so these materials present a particularly promising phase space in which to discover and study new orbitally molecular species.

We have recently reported an unprecedented mechanism of electronic phase separation (EPS) in the 6H-perovskite Ba_3_SrMo_2_O_9_.^22^ A single hexagonal *P*6_3_/*m* phase is observed at room temperature (*P*6_3_/*m*-Ba_3_SrMo_2_O_9_), but upon cooling below 230 K, a new and electronically distinct monoclinic *P*2_1_/*m* phase emerges (*P*2_1_/*m*-Ba_3_SrMo_2_O_9_) and coexists with the primary phase down to 1.6 K. *P*6_3_/*m*-Ba_3_SrMo_2_O_9_ features localized Mo_2_O_9_ spin dimers, while *P*2_1_/*m-*Ba_3_SrMo_2_O_9_ contains a 50:50 mixture of Mo–Mo bonded Mo_2_O_9_ orbital molecules and localized spin dimers. The observed phase separation appears to have an electronic origin related to competition between Mo–O–Mo superexchange and direct Mo–Mo bonding. This contrasts established electronic materials such as colossal magnetoresistant manganite perovskites, where EPS appears to depend on the presence of some chemical disorder.^23,24^ 6H-Ba_3_*B*′Mo_2_O_9_ compositions hence comprise a promising new system in which to study how unusual electronic states emerge from competing orbital and magnetic interactions.

Here, we report the synthesis and characterization of the new 6H-perovskite Ba_3_CaMo_2_O_9_. Our neutron scattering experiments reveal that Ba_3_CaMo_2_O_9_ undergoes a structural phase transition near 200 K, but, unlike Ba_3_SrMo_2_O_9_, no phase separation is observed down to 5 K. Magnetic susceptibility measurements, combined with our neutron scattering experiments, show that the Mo_2_O_9_ dimers adopt a nonmagnetic ground state but do not appear to have QM character. The diffraction data of both Ba_3_CaMo_2_O_9_ and Ba_3_SrMo_2_O_9_ have been further probed by distortion mode analysis to explore the structural features that dictate orbital molecule formation in this structure type.

## Experimental Section

Polycrystalline samples of Ba_3_CaMo_2_O_9_ were synthesized via a standard solid-state reaction. Stoichiometric quantities of BaCO_3_ (99.999%, Aldrich), CaCO_3_ (Purity, Aldrich), and MoO_3_ (99.5 +%, Aldrich) were ground and pressed into 13 mm pellets before being heated in air to 900 °C for 10 h. The pellets were then reground and repressed before heating under 5% H_2_/N_2_ at 1150 °C for 20 h, with one intermittent regrinding after 10 h.

X-ray diffraction (XRD) measurements were recorded on a Panalytical EMPYREAN diffractometer equipped with a Johansson monochromator (Cu Kα1 radiation, λ = 1.5406 Å). Scans were recorded for 1 h in the range of 10–100° with a 0.013° step size. High-resolution neutron powder diffraction (NPD) data were collected on a D2B diffractometer at the Institut Laue-Langevin (ILL) in Grenoble (λ = 1.59432 Å). The sample was placed in an 8 mm vanadium can and measured on heating at selected temperature intervals between 5 and 290 K. Scans were recorded for 2 h at each temperature. High-intensity NPD data were also collected on the D20 diffractometer at the ILL (λ = 2.40 Å) for 1.5 h at 5, 150, and 290 K to check for long-range magnetic ordering.

Rietveld refinements^25^ were performed using the GSAS/EXPGUI software.^26,27^ The background was modeled using a Chebyschev polynomial with 11 background coefficients, while peak profiles were modeled using a pseudo-Voigt function. Distortion mode analyses were performed using the ISODISTORT^28,29^ and AMPLIMODES^30^ programs.

DC magnetization measurements were performed on a Quantum Design SQUID magnetometer between 2 and 400 K. Data were collected after zero-field-cooling (ZFC) the sample in an applied field of 50 Oe. Heat capacity measurements were recorded on a Quantum Design PPMS from 2 to 300 K in 5 K intervals. DC resistivity measurements were also performed on a PPMS using the four-probe method on a polycrystalline bar between 4 and 300 K.

## Results

Rietveld refinement from high-resolution XRD and NPD data shows that Ba_3_CaMo_2_O_9_ crystallizes in the hexagonal *P*6_3_/*mmc* space group at room temperature (RT). The fit to the *P*6_3_/*mmc* model at 290 K from NPD data collected on D2B is shown in Figure S1, while selected crystallographic data are reported in Table S1. An excellent fit to the *P*6_3_/*mmc* structural model (Figure 1a) is observed, and there is no sign of any impurity phases. The *P*6_3_/*mmc* model is the typical crystal structure adopted by 6H-Ba_3_*B*′*M*_2_O_9_ compositions. Ba_3_CaMo_2_O_9_ then differs from the *B*′ = Sr analogue, which instead crystallizes in the *P*6_3_/*m* space group at 290 K due to in-phase tilting of the SrO_6_ octahedra about the hexagonal *c* axis.^22^ The atomic site occupancies could be refined to within ±1% of their nominal values and were thus fixed at 1 for the remainder of the refinements. There is no evidence of chemical disorder between the Ca and Mo sites; additionally, the large difference in ionic radii between Ca^2+^ and Mo^5+^ (1.00 Å vs 0.61 Å, respectively^31^) makes this highly unlikely. Selected bond distances and angles at 290 K are reported in Table S2. Bond valence sum (BVS) calculations gave a value of BVS(Mo) = 5.11(1), which is in good agreement with the nominal +5 oxidation state.

Variable-temperature NPD measurements recorded on D2B confirmed that Ba_3_CaMo_2_O_9_ retains the *P*6_3_/*mmc* structure down to 230 K. Below 200 K, a structural phase transition occurs as evidenced by the appearance of new peaks in the diffraction pattern. It was not possible to obtain a good fit to the previously reported structural models for 6H-perovskites, such as *Cmcm* for Ba_3_CoRu_2_O_9_^16^ or *C*2/*c* for Ba_3_CaIr_2_O_9_.^32^ Attempts to perform Rietveld fits with all direct subgroups of *P*6_3_/*mmc* were also unsuccessful. However, very good fits were obtained to two structural models described by the *Pnma* and *P*2_1_/*m* space groups (*R*_wp_ = 4.34 and 4.28% for *Pnma* and *P*2_1_/*m*, respectively, at 5 K). We found no evidence of any intermediate phase transition that might favor one of these two options, and close examination of the diffraction patterns found that the *P*2_1_/*m* model offered no tangible improvement to the peak positions, intensities, or profiles. Ultimately then, *Pnma* was selected as the correct space group assignment.

**Figure 2:**
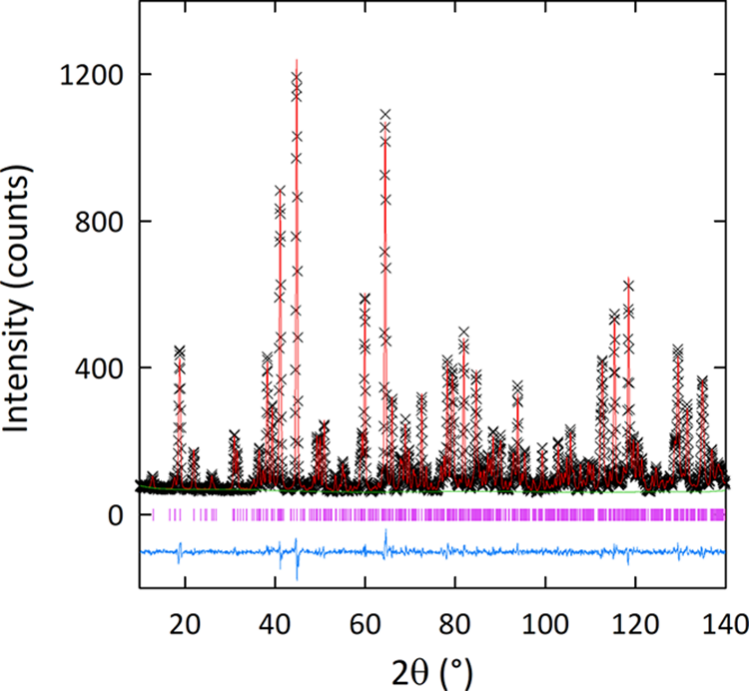
Rietveld fit to the *Pnma* structural model from NPD data collected on D2B at 5 K for Ba_3_CaMo_2_O_9_.

The Rietveld fit and crystallographic data for the *Pnma* structural model at 5 K are provided in Figure 2 and Table S3. Selected bond lengths and angles for the *Pnma* phase at this temperature are reported in Table S4. The *Pnma* structure (Figure 1b) accounts for tilting of the CaO_6_ octahedra along the orthorhombic [001] and [101] directions but does not generate any additional Ca or Mo crystallographic sites. This is in contrast to *P*2_1_/*m*-Ba_3_SrMo_2_O_9_, in which there are two crystallographically inequivalent Sr and Mo sites. The *Pnma* structure is retained down to 5 K.

**Figure 3:**
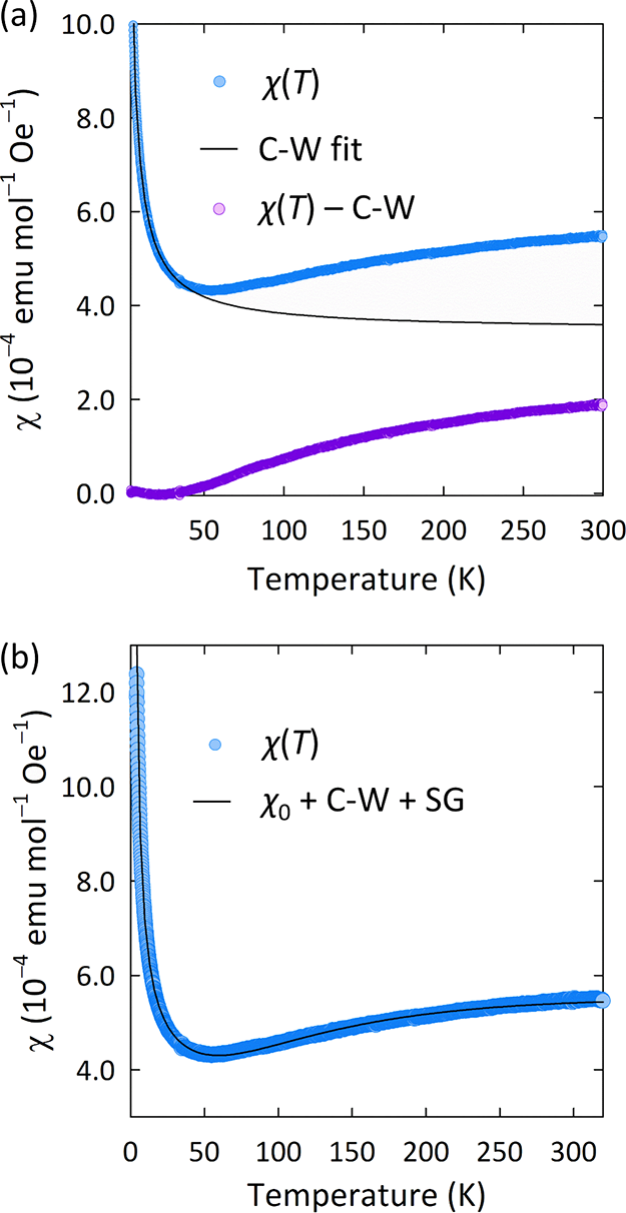
(a) Variable-temperature magnetic susceptibility (χ(*T*)) measurements of Ba_3_CaMo_2_O_9_. The initial Curie–Weiss (C-W) fit is shown by the solid black line, and its subtraction from χ(*T*) is shown in purple. (b) Fit to the spin-gap function listed in the main text, with additional temperature-independent (χ_0_) and C-W terms.

No magnetic diffraction peaks were detected on the high-intensity D20 diffractometer down to 5 K. Ba_3_CaMo_2_O_9_ was too resistive to measure from our DC resistivity measurements, showing that it is an insulator below 300 K. Heat capacity measurements confirmed that the structural transition occurs at 220 K (Figure S2). Variable-temperature magnetic susceptibility (χ(*T*)) measurements (Figure 3) corroborated the lack of long-range magnetic ordering down to 4 K so that the structural transition at 220 K is not tied to magnetic ordering of the Mo^5+^ moments. At low temperatures, a paramagnetic Curie tail is observed, while above 44 K, χ(*T*) weakly increases as a function of temperature. A small Curie constant of 3.55(2) × 10^–3^ emu K Oe^–1^ mol^–1^ was extracted from an initial Curie–Weiss fit of the data below 44 K (shown in Figure 3a); we attribute this to minor magnetic impurities or structural defects in the sample. A subtraction of the Curie–Weiss contribution from χ(*T*) shows that the residual magnetic contribution tends to zero with cooling. This response is reminiscent of spin-gapped materials such as La_4_Ru_6_O_19_^33^ or SrCu_2_O_3_^34^ and shows that Mo_2_O_9_ dimers in Ba_3_CaMo_2_O_9_ have a spin-gapped nonmagnetic ground state.

Prior to the fit, a diamagnetic correction was performed. To fit the full susceptibility response χ(*T*), we used a modified spin gap expression of the form χ_SG_ = *a*(*T*)^*–*1/2^ exp(−Δ_*x*_/*k*_B_*T*)^35^ (Figure 3b). This expression accounts for spin-gapped Mo_2_O_9_ dimers in which there is a gap between a nonmagnetic singlet state and a triplet excited state. Similar functions have been used to model spin gaps in previous 6H-perovskites such as Ba_3_BiRu_2_O_9_^36,37^ and Ba_3_BiIr_2_O_9_.^38^ In Ba_3_CaMo_2_O_9_, the spin gap expression—including additional temperature-independent and C-W terms—gives an excellent fit to χ(*T*) up to 320 K. From the fit, the following parameters were extracted: χ_0_ = 3.571(1) × 10^–4^ emu Oe^–1^ mol^–1^, *C*_imp_ = 3.35(1) × 10^–3^ emu K Oe^–1^ mol^–1^, θ = −0.23(1) K, *a* = 6.54(1) × 10^–3^ emu Oe^–1^ mol^–1^ K^–0.5^, and Δ/*k*_B_ = 233(1) K.

## Discussion

The structural and physical properties of Ba_3_CaMo_2_O_9_ are surprisingly different from Ba_3_SrMo_2_O_9_. Most notably, no phase separation is observed in Ba_3_CaMo_2_O_9_ down to 5 K as demonstrated by our high-resolution diffraction measurements. The Mo_2_O_9_ dimers are described well by a simple model of spin-gapped dimers, and there is no pronounced asymmetry in the χ(*T*) curve that would suggest that a more complex mixture of dimers forms (as previously observed for Ba_3_SrMo_2_O_9_). The properties of the spin dimers do not appear to be affected by the structural transition near 200 K, and there is also no marked lattice contraction (Figure S3). Therefore, the spin dimers in Ba_3_CaMo_2_O_9_ are not strongly coupled to the crystal lattice.

**Figure 4:**
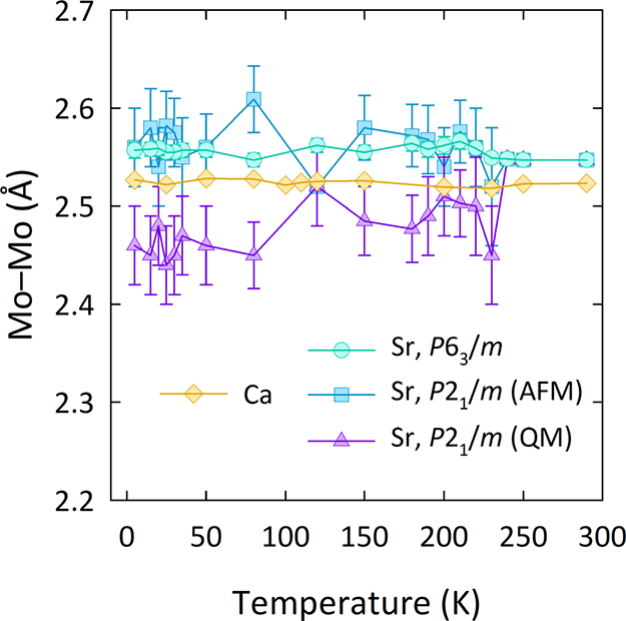
Comparison of the Mo–Mo distances in Ba_3_*B*′Mo_2_O_9_ (*B*′ = Ca and Sr^22^). “AFM” and “QM” refer to the antiferromagnetic spin dimers and quasi-molecular Mo_2_O_9_ clusters, respectively, in Ba_3_SrMo_2_O_9_.

Figure 4 compares the Mo–Mo distances in Ba_3_SrMo_2_O_9_ and Ba_3_CaMo_2_O_9_. Close inspection of the Mo–Mo distances in the Ca analogue (Figure S4) reveals that no Mo–Mo bond forms upon cooling. The Mo–Mo separation in Ba_3_CaMo_2_O_9_ is 2.523(3) Å at 290 K and remains largely similar with cooling so that the Mo–Mo separation is 2.527(3) Å at 5 K. This is in contrast to the QM Mo_2_O_9_ dimers in *P*2_1_/*m*-Ba_3_SrMo_2_O_9_, where the Mo–Mo distances contract by ∼4% below 230 K due to the formation of a Mo–Mo bond. The Mo *t*_2g_ orbitals in Ba_3_CaMo_2_O_9_ are hence too far apart to hybridize and form a quasi-molecular electronic state. Competition between Mo–Mo bonding and Mo–O–Mo superexchange is therefore less pronounced in Ba_3_CaMo_2_O_9_ than in Ba_3_SrMo_2_O_9_.

**Figure 5:**
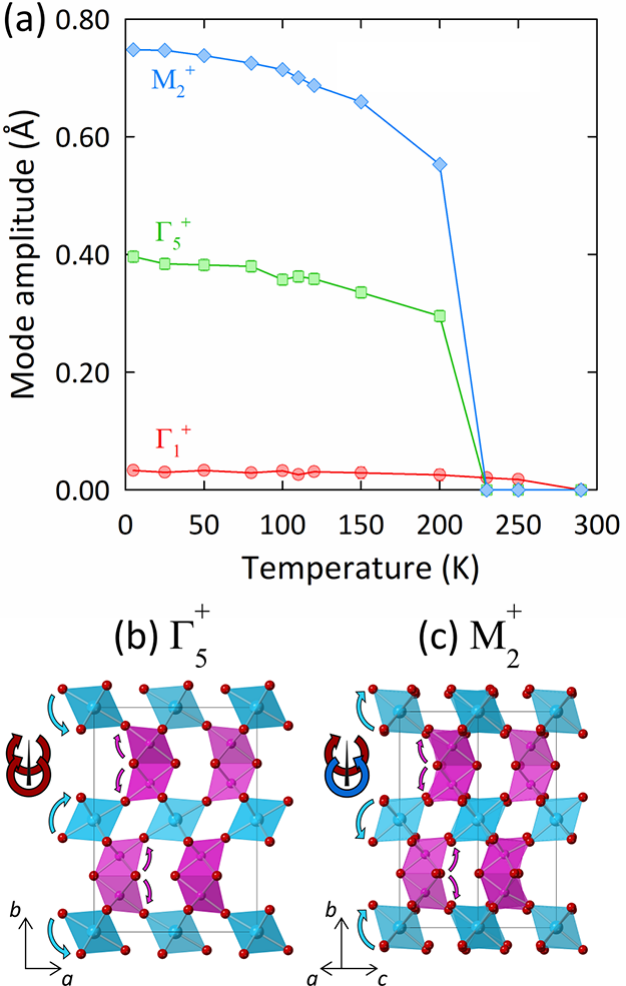
Distortion mode analysis of the structural transition to the *Pnma* phase in Ba_3_CaMo_2_O_9_. (a) Variable-temperature mode amplitudes, normalized with respect to the parent *P*6_3_/*mmc* structure; error bars are too small to be visible. (b) Γ_5_^+^ distortion mode, corresponding to an in-phase rotation of the CaO_6_ octahedra about the orthorhombic [001] direction. (c) Μ_2_^+^ distortion mode, corresponding to an anti-phase rotation of the CaO_6_ octahedra about the orthorhombic [101] direction. The arrows in panels (b, c) highlight the rotations of the CaO_6_ octahedra and Mo_2_O_9_ dimers.

Due to the differing structural and magnetic properties of Ba_3_CaMo_2_O_9_ and Ba_3_SrMo_2_O_9_, we explored sources of structural coupling in these materials that may influence whether localized spin dimers are obtained over quasi-molecular clusters. We performed distortion mode analyses using the ISODISTORT^28,29^ and AMPLIMODES^30^ programs to examine the behavior of structural degrees of freedom in both compositions. Distortion modes comprise symmetry-adapted linear combinations of irreducible representations that relate a high-symmetry crystal structure to a lower-symmetry counterpart. In particular, distortion modes describe symmetry-related sets of collective atomic displacements that represent distinct structural degrees of freedom (for example, octahedral rotations or ferroelectric polarizations). Each mode is quantified by an amplitude that describes the magnitude of the deviation from the high-symmetry structure and denotes its contribution to the total distortion observed. Hence, the advantage of this approach is that it can identify and characterize distinct structural degrees of freedom that produce a structural distortion. For Ba_3_CaMo_2_O_9_, the amplitudes of each distortion mode were derived and normalized with respect to the parent *P*6_3_/*mmc* unit cell. Three distortion modes are active in the low-temperature *Pnma* structure, corresponding to the irreducible representations Γ_1_^+^, Γ_5_^+^, and Μ_2_^+^. Their amplitudes are shown in Figure 5a, while the effects of the Γ_5_^+^ and Μ_2_^+^ modes on the crystal structure are depicted in Figure 5b and Figure 5c, respectively. The Γ_1_^+^ mode is a minor strain mode resulting from the overall compression of the unit cell along *c* with cooling. The Γ_1_^+^ mode shows no abrupt variation across the structural transition and is not associated with any octahedral tilting distortion. The Γ_5_^+^ mode consists of an in-phase tilting distortion of the CaO_6_ octahedra about the orthorhombic [001] direction, while the Μ_2_^+^ mode consists of an out-of-phase tilt of the CaO_6_ octahedra about the [101] direction. Overall, these tilting modes serve to “buckle” the Mo_2_O_9_ dimers; despite this disruption to the local MoO_6_ octahedral environment below the structural transition, the lack of any accompanying change in the magnetic susceptibility (Figure 3b) shows that the magnetic properties of the Mo_2_O_9_ dimers are largely insensitive to the structural distortion. As this distortion does not appear to be tied to any specific electronic instability, it is instead driven by purely structural fluctuations. This substantiates the lack of Mo–Mo bond formation at the transition and ultimately reflects the localized character of the Mo_2_O_9_ spin dimers. This is a notable contrast to Ba_3_SrMo_2_O_9_, where distinct changes in the magnetic susceptibility can be identified at the phase separation temperature^22^ so that the magnetic and electronic properties of the dimers are more sensitive to the distortion of the local MoO_6_ environment.

**Figure 6:**
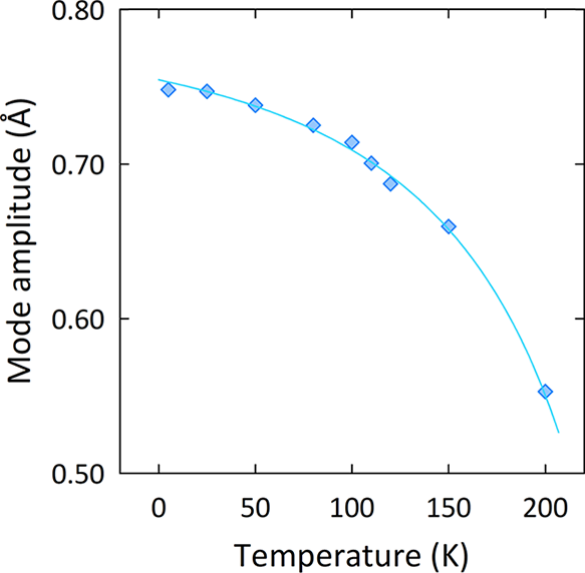
Temperature dependence of the Μ_2_^+^ distortion mode in Ba_3_CaMo_2_O_9_. The solid cyan line depicts the fit to the order-parameter-like expression given in the main text.

In Ba_3_CaMo_2_O_9_, the Μ_2_^+^ mode forms the dominant contribution to the observed distortion. Analysis of the group–subgroup relations between the *P*6_3_/*mmc* and *Pnma* space groups confirms that the Μ_2_^+^ mode forms the primary order parameter of the transition as it is the only mode capable of producing the final *Pnma* symmetry. *Pnma* is not a direct subgroup of *P*6_3_/*mmc*, so the transition is necessarily first order. Despite this, the unit cell volume shows no clear contraction upon cooling below the phase transition temperature (Figure S5), which would appear to be inconsistent with a first-order transition. However, Figure 6 shows that the Μ_2_^+^ mode amplitudes (*X*) can be fit to a critical equation of the following form:
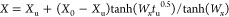
1where *X*_u_ and *X*_0_ are the amplitudes at the transition temperature (*T*_u_) and 0 K, respectively, *W_x_* is a fitting parameter (*W* ≈ 2), and *t*_u_ is the reduced temperature defined as *t*_u_ = (*T*_u_ – *T*)/*T*_u_. This empirical expression describes the critical variation of order-parameter-like structural quantities below a phase transition.^39^Equation 1 is particularly successful in describing the variations of such quantities at temperatures well below the transition temperature (*T* ≪ *T*_u_); hence, it can be used to model the behavior of distortion modes across extended temperature ranges. Equation 1 has been used previously to model the behavior of structural quantities that vary as order parameters in materials featuring quasi-molecular clusters such as Fe_3_O_4_^39^ and GaV_2_O_4_.^40^ Here, a transition temperature of *T*_u_ = 225 K was extracted from the fit (Figure 6), which is in excellent agreement with the temperature determined from our heat capacity measurements. The parameters *X*_0_ and *X*_u_ can also be compared to characterize the behavior of the transition, that is, whether the structural distortion is largely frozen below *T*_u_ (*X*_u_/*X*_0_ ≈ 1), or whether it is quasi-continuous in character (*X*_u_/*X*_0_ ≈ 0).^39^ Frozen transitions feature largely temperature-invariant structural changes below *T*_u_, whereas quasi-continuous transitions display more temperature-dependent behavior. In the case of Ba_3_CaMo_2_O_9_, the ratio *X*_u_/*X*_0_ ≈ 0.45 tends toward the quasi-continuous limit over the frozen limit. This explains the lack of any notable discontinuity in unit cell volume upon cooling below the structural transition. It appears then that thermal effects largely dictate the structural behavior of Ba_3_CaMo_2_O_9_, and the transition does not appear to be tied to any specific electronic instability. This substantiates the lack of Mo–Mo bond formation at the transition and ultimately reflects the localized character of the Mo_2_O_9_ spin dimers.

**Figure 7:**
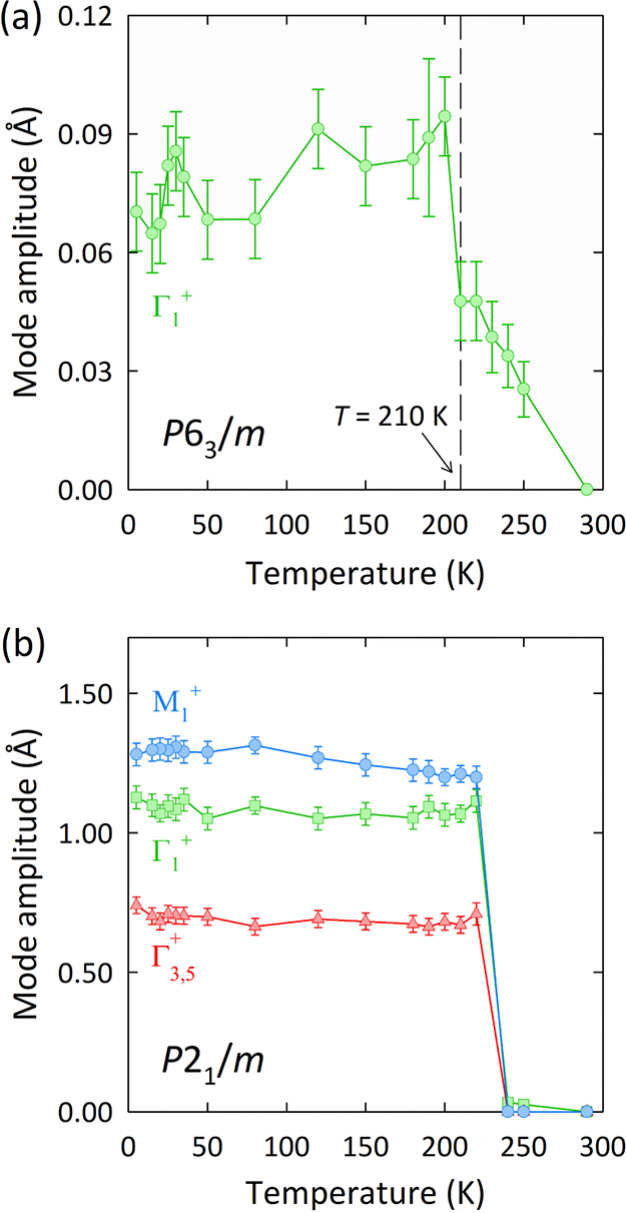
Distortion mode analyses of the (a) *P*6_3_/*m* and (b) *P*2_1_/*m* phases of Ba_3_SrMo_2_O_9_. Mode amplitudes were derived and normalized with respect to their respective parent unit cells. Where not apparent, error bars are smaller than the data points. Data for the *P*2_1_/*m* phase at 230 K have been omitted as the small phase fraction at this temperature did not allow for a reliable quantification of the mode amplitudes.

We performed a comparative distortion mode analysis on Ba_3_SrMo_2_O_9_ based on our previously reported NPD data.^22^ The distortion mode amplitudes for both phases of Ba_3_SrMo_2_O_9_ are shown in Figure 7. Here, the distortion modes have been labeled with respect to the parent *P*6_3_/*m* symmetry as the *P*6_3_/*mmc* phase has not been reported for Ba_3_SrMo_2_O_9_. For the *P*6_3_/*m* phase, the low-temperature distortion is described by a single Γ_1_^+^ mode. Predominantly, the Γ_1_^+^ mode corresponds to in-phase tilting of the SrO_6_ octahedra and Mo_2_O_9_ dimers about the hexagonal *c* axis; in 6H-perovskites, this distortion has been canonically attributed to the size mismatch between the *B*′ and *M* sites.^41^ The amplitude of the Γ_1_^+^ mode in *P*6_3_/*m*-Ba_3_SrMo_2_O_9_ shows a discontinuous increase below 210 K (Figure 7a). Interestingly, this discontinuity overlaps with a previously identified lattice anomaly in this phase, where there is an abrupt contraction of the lattice along *c* below 210 K without any accompanying structural transition.^22^ We previously showed that the phase fractions of the *P*6_3_/*m* and *P*2_1_/*m* phases in Ba_3_SrMo_2_O_9_ were sensitive to this lattice anomaly: the weight fraction of the *P*2_1_/*m* phase attains a maximum of 47.6% at 210 K, but this fraction gradually decreases below this temperature so that the lattice anomaly promotes the *P*6_3_/*m* phase instead. The distortion stemming from the Γ_1_^+^ mode is then associated with the anomalous lattice contraction in this phase. We also note that the amplitude of this mode correlates with the Mo–O(1)–Mo bond angles (Figure S6), where the Mo–O(1)–Mo bond angles tend toward the ideal angle for magnetic superexchange interactions. This structural correlation suggests that the lattice anomaly serves to promote intradimer superexchange pathways in *P*6_3_/*m*-Ba_3_SrMo_2_O_9_ so that this is the mechanism by which the *P*6_3_/*m* phase becomes favored over the *P*2_1_/*m* phase upon further cooling.

For *P*2_1_/*m*-Ba_3_SrMo_2_O_9_, there are three distortion modes active in the low-temperature regime. The Γ_1_^+^ and Γ_3,5_^+^ modes predominantly correspond to in-phase tilting distortions of the SrO_6_ octahedra about the pseudohexagonal *c* and *a* axes, respectively, while the M_1_^+^ distortion mode corresponds to an out-of-phase tilt of the SrO_6_ octahedra about the pseudohexagonal *b* axis. The structural distortions in Ba_3_SrMo_2_O_9_ and its Ca analogue are hence largely similar, with the notable exception of the SrO_6_ tilting associated with the Γ_1_^+^ mode in Ba_3_SrMo_2_O_9_. The M_1_^+^ mode comprises the dominant contribution to the structural distortion in *P*2_1_/*m*-Ba_3_SrMo_2_O_9_ at lower temperatures (Figure 7b) so that it is the primary order parameter for the transition. Although the Γ_1_^+^ mode also has a significant amplitude in this phase, its amplitude shows no apparently critical variation with cooling. This suggests that this mode behaves as a secondary order parameter so that it is coupled to the primary M_1_^+^ distortion. In contrast, the M_1_^+^ mode shows an overall increase upon cooling (Figure S7). The temperature variation of the M_1_^+^ amplitude can be fit with the critical expression given in eq 1, as shown in Figure S8. A transition temperature of *T*_u_ = 241 K was extracted from the fit, which is in agreement with the experimental temperature previously identified from neutron diffraction and heat capacity measurements. The transition is also characterized by the ratio *X*_u_/*X*_0_ = 0.80, suggesting that Ba_3_SrMo_2_O_9_ tends toward the frozen limit over the quasi-continuous limit.

**Figure 8:**
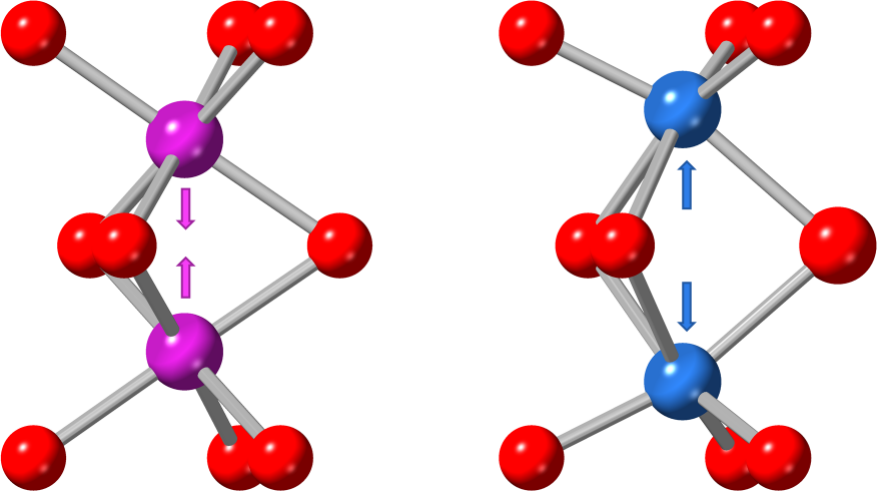
Asymmetric M_1_^+^ [Mo:f]A(a) displacement in Ba_3_SrMo_2_O_9_. Quasi-molecular dimers are shown in pink, while the localized spin dimers are shown in blue.

Closer examination of the individual atomic displacements for Ba_3_SrMo_2_O_9_ reveals a subtle distinction to its *B*′ = Ca counterpart. The M_1_^+^ mode in *P*2_1_/*m*-Ba_3_SrMo_2_O_9_ changes the intradimer Mo–Mo distances according to the [Mo:f]A(a) displacement (Figure 8). The two crystallographically distinct Mo_2_O_9_ dimers in this phase feature an antiferrodistortive displacement of the Mo atoms along *c* so that they displace toward each other within the QM clusters and away from each other within the AFM spin dimers. In contrast, the displacement of the Mo atoms in Ba_3_CaMo_2_O_9_ is limited by symmetry to the Μ_2_^+^ [Mo:f]E(a) distortion, which corresponds to an antiferrodistortive displacement within the pseudohexagonal basal plane; as the intradimer Mo–Mo distances lie parallel to the crystallographic long axis, there is hence no contraction of the intradimer Mo–Mo distances in Ba_3_CaMo_2_O_9_. Mo–Mo bonding is therefore strongly coupled to the crystal lattice in *P*2_1_/*m*-Ba_3_SrMo_2_O_9_, and the near-frozen character of the structural transition in this phase reflects the crystallization of the QM dimer network. In contrast, there appears to be no such behavior in Ba_3_CaMo_2_O_9_ so that orbital molecules do not form in this composition and the electrons remain entangled in a localized spin state. It is likely that the more covalent Ca–O bond draws electron density away from the Mo_2_O_9_ dimers so that Mo–Mo bonding is less favored than in the *B*′ = Sr counterpart.

## Summary

Ba_3_CaMo_2_O_9_ is a novel 6H-perovskite featuring localized Mo_2_O_9_ spin dimers below 320 K. A structural transition is observed near 200 K, but the electronic and magnetic properties of the Mo_2_O_9_ dimers appear to be largely insensitive to the resulting distortion so that the transition does not produce Mo–Mo bonding. Our distortion mode analyses suggest that tilting of the *B*′O_6_ octahedra along *c* in 6H-perovskites is correlated with the intradimer superexchange pathways. Size mismatch between the *B*′ and *M* sites could hence constitute an appropriate chemical parameter to tune intradimer exchange interactions and discover new ground states in new or existing 6H-perovskites. This approach could also be extended to other materials featuring orbital molecules to probe the sensitive interplay between bonding interactions and magnetic exchange.

## Supporting Information Available

Figures showing Rietveld refinement fits, tables of crystallographic data, and figures showing the temperature variation of the heat capacity, cell parameters and volume, and mode amplitudes (PDF)
