# Supplementary material for: Localized Spin Dimers and Structural Distortions in the Hexagonal Perovskite Ba3CaMo2O9
Source: Inorg Chem. 2022 Jul 19;61(30):11622–8. doi: 10.1021/acs.inorgchem.2c01102 (PMC9377418; doi:10.1021/acs.inorgchem.2c01102)
Supplement: Supplementary file 1 — ic2c01102_si_001.pdf [file ic2c01102_si_001.pdf]

Supplementary Information

for

Localised spin dimers and structural distortions in the hexagonal perovskite $Ba_3CaMo_2O_9$

Struan Simpson[1], Michael Milton[1], Sacha Fop[1], Gavin B. G. Stenning[2], Harriet Alexandra Hopper[1], Clemens Ritter[3], and Abbie C. Mclaughlin[1]*

[1] *Chemistry Department, University of Aberdeen, Meston Walk, Aberdeen, AB24 3UE, U.K.*

[2] *ISIS Experimental Operations Division, Rutherford Appleton Laboratory, Harwell Science and Innovation Campus, Didcot, OX11 0QX, U.K.*

[3] *Institut Laue Langevin, 71 Avenue des Martyrs, BP 156, F-38042 Grenoble Cedex 9, France.*

*a.c.mclaughlin@abdn.ac.uk

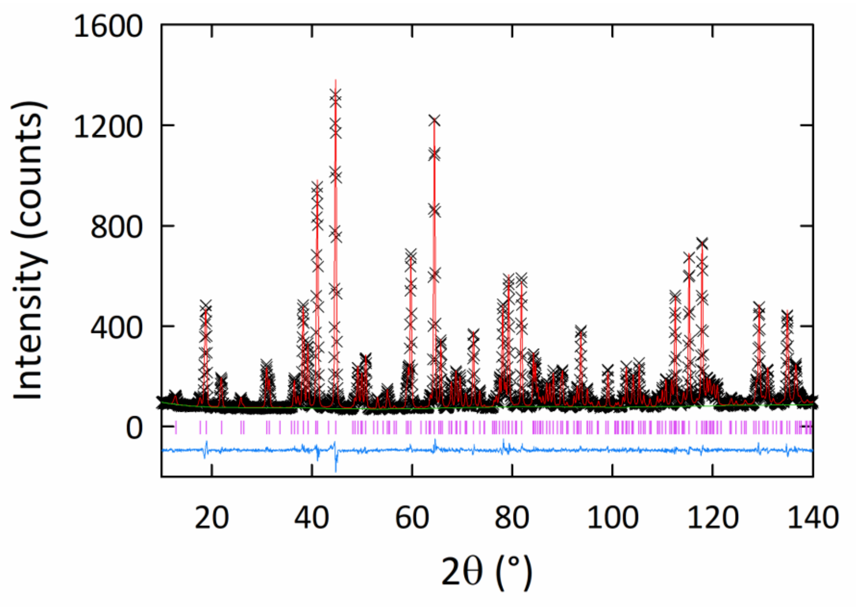

Figure S1. The *P*6$_3$/*mmc* Rietveld fit to 290 K NPD data collected on D2B for $Ba_3CaMo_2O_9$.

Table S1. Refined atomic parameters obtained from the 290 K *P*6$_3$/*mmc* Rietveld fit. U_{ij} denotes anisotropic displacement parameters in units of Å^2. a = 5.9828(1) Å, c = 14.2785(1) Å, R_{wp} = 4.02%, R_p = 3.12%, χ^2 = 2.14.

Atom	Site	*x*	*y*	*z*	U_{11}	U_{22}	U_{33}	U_{12}	U_{13}	U_{23}
Ba(1)	2*b*	0	0	¼	0.0092(9)	0.0092(9)	0.007(2)	0.0047(5)	0	0
Ba(2)	4*f*	⅓	⅔	0.0987(1)	0.0061(6)	0.0061(6)	0.007(1)	0.0031(3)	0	0
Ca	2*a*	0	0	0	0.0012(8)	0.0012(8)	0.006(2)	0.0006(4)	0	0
Mo	4*f*	⅓	⅔	0.8384(1)	0.0038(4)	0.0038(4)	0.005(1)	0.0019(2)	0	0
O(1)	6*h*	0.5088(2)	0.0177(3)	¼	0.0204(6)	0.0027(8)	0.006(1)	0.0014(4)	0	0
O(2)	12*k*	0.8220(1)	0.6440(2)	0.0925(1)	0.0170(5)	0.0086(6)	0.017(1)	0.0043(3)	0.0045(2)	0.0090(4)

Table S2. Selected interatomic distances and angles for the $P6_3/mmc$ phase at 290 K.

Distance (Å)		Angle (°)	
Ca–O(2) x 6	2.2689(1)	O(1)–Mo–O(1) x 3	86.58(6)
Mo–O(1) x 3	2.066(2)	O(2)–Mo–O(2) x 3	95.18(6)
Mo–O(2) x 3	1.888(1)	O(1)–Mo–O(2) x 3	173.86(8)
Mo–Mo x 2	2.523(3)	Mo–O(1)–Mo	75.30(8)

Table S3. Refined atomic parameters obtained from the 5 K *Pnma* Rietveld fit. a = 10.3553(2) Å, b = 14.2080(2), c = 5.9855(1) Å, R_{wp} = 4.34%, R_p = 3.41%, χ^2 = 2.31.

Atom	Site	*x*	*y*	*z*	U_{iso} (Å^2)
Ba(1)	4*c*	–0.0102(7)	1/4	0.0222(9)	0.0034(8)
Ba(2)	8*d*	0.3304(5)	0.0997(1)	–0.0013(7)	0.0024(4)
Ca	4*a*	0	0	0	0.0027(6)
Mo	8*d*	0.3384(4)	0.8389(1)	0.0074(5)	0.0034(4)
O(1_1)	4*c*	0.0046(6)	1/4	0.4736(10)	0.0074(13)
O(1_2)	4*c*	0.2652(5)	1/4	0.7145(9)	0.0064(10)
O(1_3)	4*c*	0.2642(5)	3/4	0.7616(9)	0.0027(10)
O(2_1)	8*d*	0.3145(4)	0.0867(3)	0.4842(7)	0.0074(8)
O(2_2)	8*d*	0.0809(3)	0.0844(3)	0.2840(6)	0.0063(7)
O(2_3)	8*d*	0.4131(4)	0.8935(2)	0.2554(5)	0.0061(6)

Table S4. Selected interatomic distances and angles for the *Pnma* phase at 5 K.

Distance (Å)		Angle (°)	
Ca–O(2_1) x 2	2.283(4)	O(2_1)–Ca–O(2_2) x 2	90.3(1)
Ca–O(2_2) x 2	2.243(4)	O(2_1)–Ca–O(2_3) x 2	90.0(1)
Ca–O(2_3) x 2	2.290(3)	O(2_2)–Ca–O(2_3) x 2	89.1(1)
Mo–O(1_1) x 2	2.069(7)	Mo–O(1_1)–Mo	75.3(3)
Mo–O(1_2) x 2	2.070(5)	Mo–O(1_2)–Mo	75.2(2)
Mo–O(1_3) x 2	2.086(5)	Mo–O(1_3)–Mo	74.6(2)
Mo–O(2_1) x 2	1.909(5)	O(1_1)–Mo–O(1_2)	89.8(2)
Mo–O(2_2) x 2	1.916(5)	O(1_1)–Mo–O(1_3)	100.0(2)
Mo–O(2_3) x 2	1.845(4)	O(1_2)–Mo–O(1_3)	98.0(2)
Mo–Mo	2.527(3)		

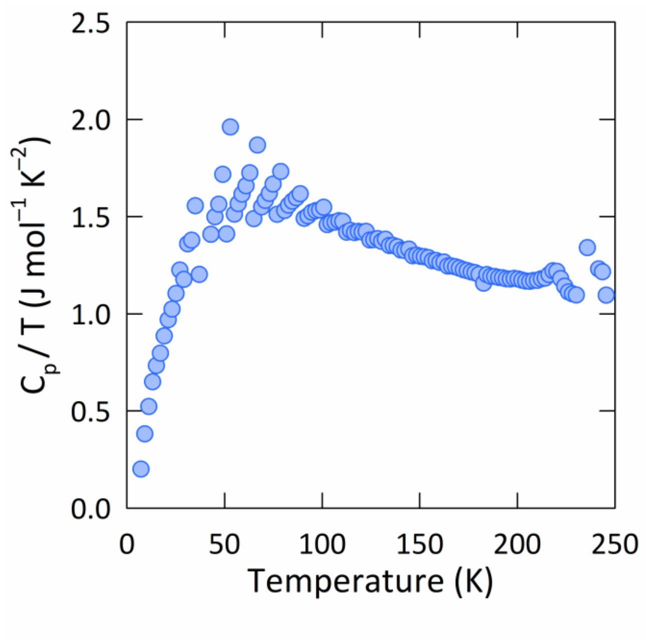

Figure S2. Heat capacity (C_p/T) measurements performed on $Ba_3CaMo_2O_9$, showing a notable peak in the C_p/T ratio centred at 220 K. The data below 150 K become increasingly noisy due to poor thermal coupling between the sample and the sample platform.

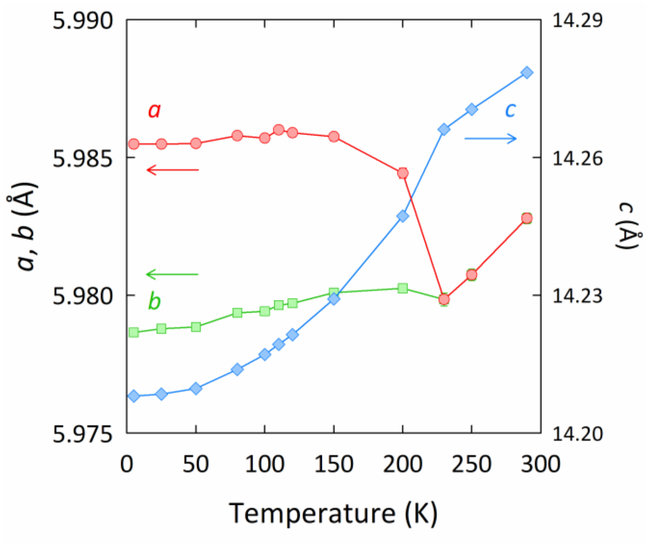

Figure S3. Variation in cell parameters with temperature obtained from Rietveld refinement of NPD data. Cell parameters have been normalised and labelled with respect to the parent $P6_3/mmc$ structure for consistency. Where not apparent, error bars are smaller than the data points.

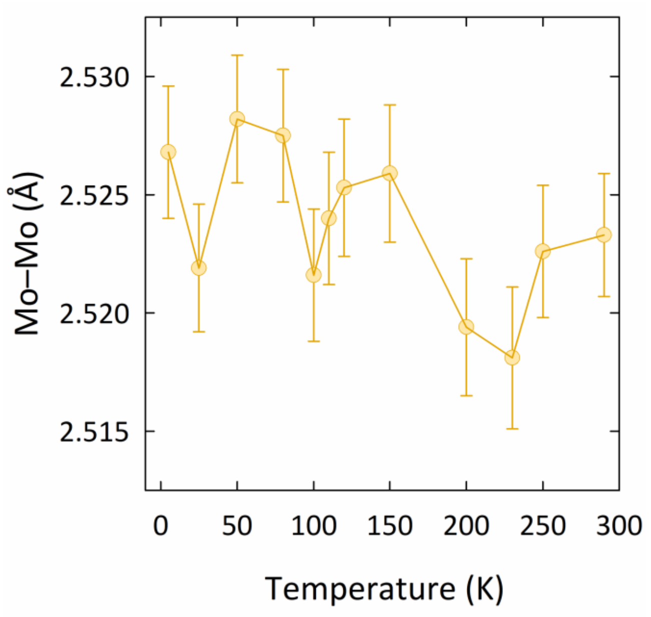

Figure S4. Variation in the Mo–Mo distance with temperature, showing no consistent trend or change with temperature.

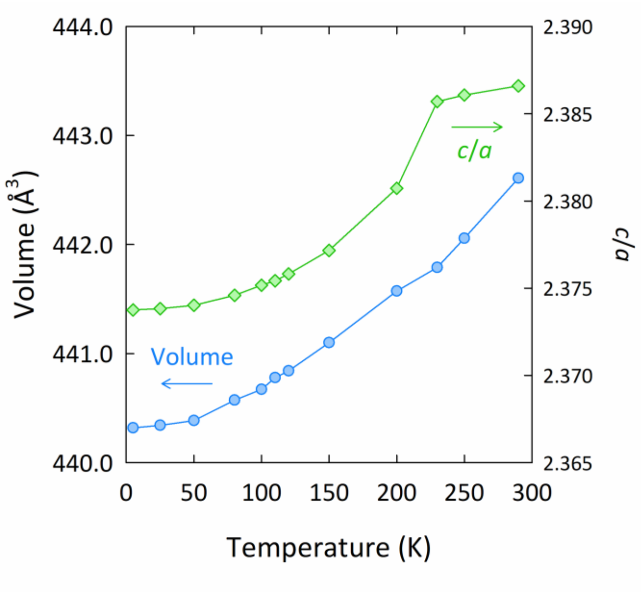

Figure S5. Temperature dependences of the unit cell volume and *c*/*a* ratio. Error bars are smaller than the data points.

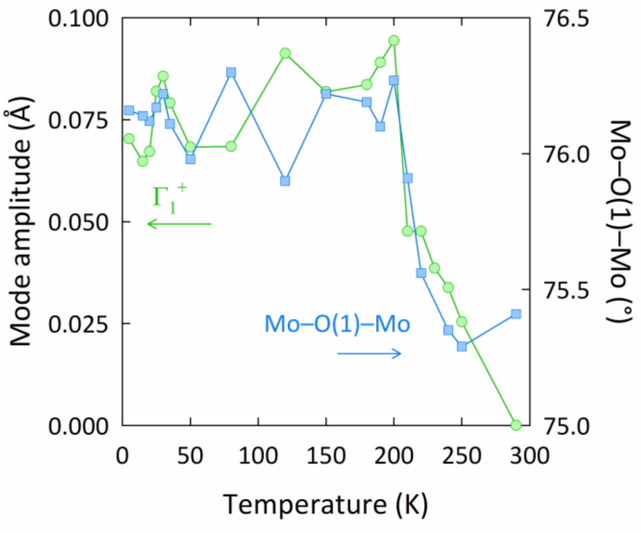

Figure S6. Correlation between the Γ^{1+} mode amplitude and the Mo–O(1)–Mo bond angle in $P6_3/m$-$Ba_3SrMo_2O_9$, showing the overall increase in both below 210 K.

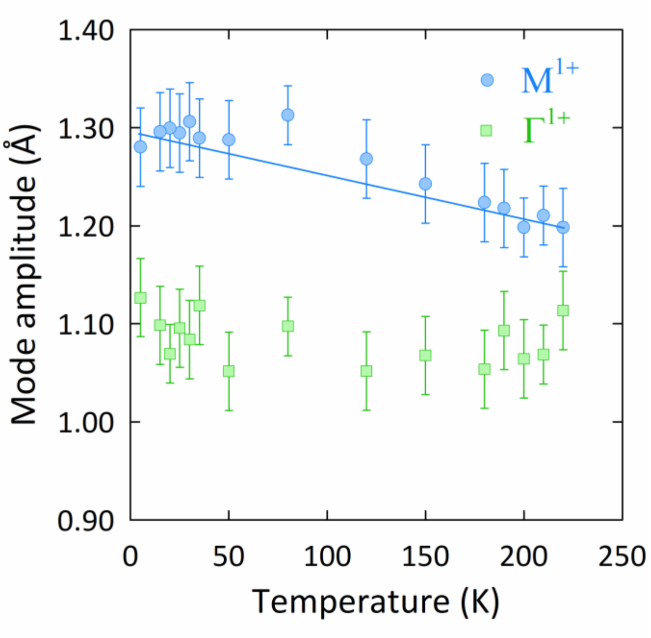

Figure S7. Temperature dependence of the Γ^{1+} and M^{1+} distortion modes for $P2_1/m$-$Ba_3SrMo_2O_9$. The solid blue line for the M^{1+} mode is shown for visual guidance to highlight the overall increase in the M^{1+} distortion amplitude with cooling.

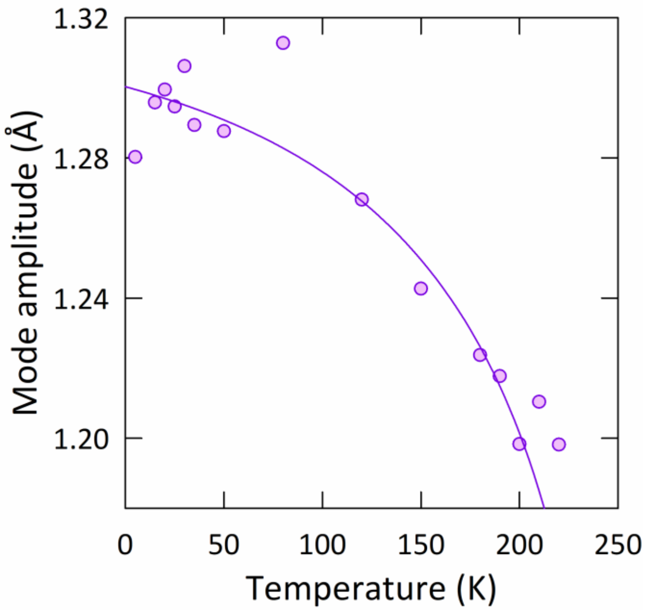

Figure S8. Fit of the M^{1+} mode amplitude to Equation 1 for $P2_1/m$-$Ba_3SrMo_2O_9$. The solid purple line denotes the fit.

Table S5 Crystallographic data table

**Source**	Neutron (constant wavelength)
**Chemical formula**	$Ba_3CaMo_2O_9$
**Formula weight**	835.4956
**Temperature (K)**	290
**Crystal System**	Tetragonal
**Space group**	$P6_3/mmc$ (no. 194)
***a* (Å)**	5.9828(1)
***c* (Å)**	14.2785(1)
**V (Å^3)**	442.61(1)
Z	2
***d*-space range (Å)**	0.85 – 9.2
χ^2	2.14
**R_P**	3.12
**R_{WP}**	4.02
**Definition of R factors**	$R_p = \Sigma\|y_i(obs) - y_i(calc)\|/\Sigma y_i(obs)$; $R_{wp} = \{\Sigma w_i[y_i(obs) - y_i(calc)]^2/\Sigma w_i y_i(obs)^2\}^{1/2}$
